# Artificial intelligence applied in acute ischemic stroke: from child to elderly

**DOI:** 10.1007/s11547-023-01735-1

**Published:** 2023-10-25

**Authors:** Francesco Pacchiano, Mario Tortora, Sabrina Criscuolo, Katya Jaber, Pasquale Acierno, Marta De Simone, Fabio Tortora, Francesco Briganti, Ferdinando Caranci

**Affiliations:** 1 Department of Precision Medicine, University of Campania “L. Vanvitelli”, Caserta, Italy; 2 Department of Advanced Biomedical Sciences, University “Federico II”, Via Pansini, 5, 80131 Naples, Italy; 3 Pediatric University Department, Bambino Gesù Children Hospital, Rome, Italy; 4 Department of Elektrotechnik und Informatik, Hochschule Bremen, Bremen, Germany; 5 UOC Neuroradiology, AORN San Giuseppe Moscati, Avellino, Italy

**Keywords:** Stroke, Artificial intelligence, Interventional neuroradiology, Acute stroke therapy, Ischemic stroke

## Abstract

This review will summarize artificial intelligence developments in acute ischemic stroke in recent years and forecasts for the future. Stroke is a major healthcare concern due to its effects on the patient’s quality of life and its dependence on the timing of the identification as well as the treatment. In recent years, attention increased on the use of artificial intelligence (AI) systems to help categorize, prognosis, and to channel these patients toward the right therapeutic procedure. Machine learning (ML) and in particular deep learning (DL) systems using convoluted neural networks (CNN) are becoming increasingly popular. Various studies over the years evaluated the use of these methods of analysis and prediction in the assessment of stroke patients, and at the same time, several applications and software have been developed to support the neuroradiologists and the stroke team to improve patient outcomes.

## Introduction

Stroke is a common cause of morbidity and mortality around the world. Between 1990 and 2019, the burden (measured in terms of the total number of cases) increased significantly (incident strokes increased by 70.0% and stroke deaths increased by 43.0%). One in four people over the age of 25 will experience a stroke in their lifetime, and there are over 12.2 million new cases of stroke each year worldwide. The most prevalent type of stroke, the ischemic subtype (AIS), accounts for more than 62% of all occurrences worldwide [1]. The first diagnostic test performed when a stroke is suspected is a CT scan without contrast medium (NCCT), which enables us to detect intracerebral hemorrhage (ICH) and assess parenchymal ischemia symptoms like decreased differentiation between white and gray matter [2]. Quantifying ischemic damage and determining whether a patient is a candidate for treatment are made possible by the Alberta stroke program early CT score (ASPECTS) [3]. The detection of a large vessel occlusion (LVO) and evaluation of collateral vessels are both possible with CT angiography (CTA). We can create time density curves for each voxel in perfusion CT by measuring the density change brought on by the arrival of contrast medium over time. From these curves, we can derive parameters like cerebral blood volume (CBV), cerebral blood flow (CBF), and time to peak (TTP). We can evaluate the mismatch between the penumbra and necrotic core using these parameters [2]. Diffusion-weighted imaging (DWI) and apparent diffusion coefficient (ADC) on MRI are the imaging modalities with the highest sensitivity for detecting cerebral ischemia, with 73–92% sensitivity in the first 3 h and 95–100% sensitivity in the first 6 h, respectively. In situations where the time of stroke onset is unknown, such as in wake-up strokes, FLAIR/T2 weighted imaging can also offer crucial information, such as the time of occlusion. Similar to CTA, MR angiography (MRA) can detect the presence of vessel occlusion; MRA has a sensitivity of 87% and a specificity of 98% compared to CTA’s maximum sensitivity and specificity of 98% [4].

Artificial intelligence (AI) describes the creation of computer systems that can carry out tasks that would typically require human intelligence. In machine learning (ML), a branch of artificial intelligence, computer systems get smarter over time. ML algorithms can be categorized into three groups: reinforcement learning, unsupervised learning, and both. A subtype of machine learning (ML) known as neural networks imitates neurons by having layers made up of nodes. The first layer in a neural network is the input layer, followed by a variable number of hidden layers and one output layer. An artificial neural network (ANN) uses a set of parameters called weights that represent the connections between the neurons to determine how strong the connections are [5]. Backpropagation, a technique used in training, involves adjusting the weights to reduce the discrepancy between the output and the predicted output [6]. The weights are calibrated to minimize the cost function during backpropagation, which compares the output with the “ground truth” in order to determine how far off the model is. In an ANN, which can be constructed as a CNN or a recurrent neural network pattern (RNN), DL is composed of numerous hidden layers. Because it is computationally expensive to have weights connecting every neuron, CNN applies a small “kernel” or “filter” of weights at each position in the image, sliding through the image to determine the value of the neuron of the next layer.

Different kernels can be used for each layer, resulting in multiple “channels” in each layer and assisting the model in detecting features like edges and textures in our datasets. Before the output layer, the CNN layers can be connected to “fully connected” neurons, and the various kernels and weights can be adjusted using backpropagation [7].

Over the years, maximum likelihood estimation and the Bayesian method have become popular statistical tools applied to machine learning for output validation. The asymptotic properties of both statistical methods are one of the main reasons that have increased their popularity in recent years. The difference between these two approaches is that the parameters for maximum likelihood estimation are fixed but unknown, while the parameters for the Bayesian method act as random variables with known priority distributions. Usually the Bayesian method performs better than maximum likelihood estimation in machine learning [8].

In comparison to visual inspection by human experts, these models may offer a number of advantages, including speed, large-scale deployment, objective and quantitative evaluation, and the ability to spot minute voxel-level patterns. Considerations like feature selection, classifier type, and DL are crucial when using these methods for imaging.

## Stroke classification

A major concern is determining the subtype of stroke. Even though AIS is the most prevalent subtype, identifying intracranial hemorrhage (ICH) is crucial. Several studies emphasized the ICH identifications [9, 10] but we would like to bring the focus of this article to AIS. Distinguishing the subtype of AIS turns out to be particularly challenging, in contrast to hemorrhagic stroke. Garg et al. analyzed a group of cases and established stroke subtype categorization at admission using the Trial of Org 10,172 in acute stroke treatment (TOAST) classification system using electronic health records (EHRs) and ML algorithms for natural language processing. This classification separates ischemic stroke into five different subtypes: great artery atherosclerosis, cardio-embolic small vessel occlusion, other determined etiology stroke, and stroke with unknown etiology [11]. The concordance index kappa was the highest for the cardio-embolic subtype and lowest for the cryptogenic subtype [6, 12, 13].

## Perinatal and pediatric stroke

Perinatal stroke comprises a specific group of cerebrovascular diseases that occur between 20 weeks of fetal life and 28 days postnatal life [14]. The estimated incidence is between 1:1600 and 1:3000 live births [15]. Stroke etiology is frequently poorly understood. By identifying specific prenatal stroke disease states, neuroimaging developments have aided clinical care and research growth. Neonatal periventricular venous infarction (PVI), neonatal arterial ischemia, neonatal cerebral sinus-venous thrombosis, neonatal hemorrhagic stroke, arterial presumed perinatal stroke, and presumed perinatal hemorrhagic stroke are the six distinct neonatal stroke disorders that can be distinguished based on clinical signs and neuroimaging. Hemiparetic cerebral palsy, also known as periventricular venous infarction, is primarily characterized by motor impairment. Large lesions, along with both motor and non-motor morbidities, are frequently brought on by arterial ischemic strokes in the near future [16]. About 90% of published cases are arterial ischemic variety [17]. Diffusion MRI is the gold standard for diagnosis [18]. The treatment is still debated and in most cases off-label. Anticoagulation is considered safe in pediatric patients [19], but studies focused on anticoagulation for neonatal arterial ischemic stroke are scarce. The use of steroids remains controversial but should be considered when there is evidence of arteriopathy. Acute therapy focuses on neuroprotection. Emergency recanalization strategies are precluded, because precise timing can never be known, the infarct is typically well established, and the affected artery is often open [20]. Supportive care is provided along with anti-seizure medication [21]. Stroke also affects infants (as previously described) and children. The incidence of childhood stroke has varied widely in the literature. The incidence of strokes in children ranges between 2.5 and 13:100,000 per year [22]. Stroke has a high rate of morbidity and mortality, even among children. Since it occurs at a young age and the duration of disability is longer, even lasting a lifetime. Additionally, there are significant diagnostic delays in children [23]. Pediatric strokes are linked to many diseases [24]. The most significant risk factors for stroke in children are coagulopathies, infections, vascular diseases, and cardiac causes. Head injury, autoimmune diseases, metabolic issues, child maltreatment, renal diseases, and hematological illnesses are additional risk factors [25]. Treatment for pediatric acute strokes is time-sensitive. Immediate mechanical thrombectomy or intravenous tissue plasminogen activator (tPA) therapy for children resulted in better functional and mortality outcomes. The earlier treatment begins, the greater the chance of maintaining the penumbra, restoring cerebral blood flow, and perhaps even curing the symptoms, which lessens disability. Early detection is essential for better patient care due to the narrow treatment window. It improves hospital care and lessens the chance of a stroke recurrence before recovery. Despite the lack of data and extensive randomized clinical trials, mechanical thrombectomy and intravenous tPA have both been used successfully in pediatrics [26–28].

To our knowledge, few authors have investigated the application of ML in the context of pediatric stroke; however, Carlson et al. [29]’s interesting work, which used an RF model to identify which factors might be more predictive of motor outcome in a group of 49 patients with a history of perinatal stroke (AIS and PVI), is worth mentioning. Their model included demographic information along with variables from neuroimaging using conventional MRI sequences and cutting-edge research like white matter tractography and functional MRI. A validated bimanual test known as the Assisting Hand Assessment (AHA) and a bimanual test known as the Box and Blocks Test (BBT), which was divided into two scores: one for the stroke-affected hand (BBTA) and one for the other side (BBTU), were used to assess the motor function. Twenty-seven volunteers who were roughly the same age as the comparison group made up the control group. The RRELIEFF algorithm was used to rank the features, and the RF model was then applied to model the regression for each motor outcome score.

Their research indicated that many features, including connectivity between bilateral primary motor, sensory, and supplementary areas, inter-hemispheric connectivity within the deep nuclei, and connectivity within the lesioned corticospinal tract, had lower functional and structural connectivity in children with a history of AIS. With differences in the lesioned CST and inter-hemispheric connection within the basal ganglia, it was discovered that children with a history of PVI were more similar to the control group. Enhancing our knowledge of neuroplasticity and brain remodeling after an event like a stroke may be fascinating in the future in order to create rehabilitation programs with even better results.

The use of AI software in pediatric stroke management would be desirable to optimize the management of perinatal and pediatric stroke. Even today, it seems off-label to use such software. In addition, given the pathologic variations, it would be preferable to support studies that advance knowledge of the appropriate diagnostic-therapeutic management of perinatal and pediatric stroke. To this end, it would be appropriate to start machine learning processes in order to get outcomes for adult stroke similar to those we describe below.

## Early diagnose

ASPECTS, LVOs detection, segmentation of the necrotic core and the penumbra, and diagnosis timing all play significant roles in AIS. Numerous AI programs have been created to aid in diagnosis, and numerous studies have examined how they affect ASPECTS scoring. In the event of a middle cerebral artery (MCA) occlusion, ASPECTS is a quick method to evaluate the severity of the AIS. ASPECTS has high sensitivity and specificity for both functional outcome and intracerebral hemorrhage in thrombolytic therapy, deducting one point from a value of 10 for each MCA territory involved [3]. Correct scoring is crucial when determining thrombectomy eligibility. Using RAPID software, Maegerlein et al. assessed the agreement between the ASPECTS of two seasoned neuroradiologists. Consensus readings were determined using imaging data from the baseline and follow-up CT scans conducted after six weeks. The software analysis revealed optimal agreement (*κ* ≈ 0.9), whereas the neuroradiologists’ consensus agreement was only moderate (*κ* ≈ 0.56); the neuroradiologists’ consensus agreement became comparable to the software after the 4 h time period from onset [30].

Sundaram et al. conducted a comparative analysis of concurrent CBV ASPECTS based on CTP and evaluated ASPECTS by Brainomix against a neuroradiologist assessment. Automated scores were comparable to consensus readings and CTP-CBV ASPECTS when they were grouped by the time from symptom onset (> 6 or 6 h); automated scores agreed with consensus readings and CTP-CBV ASPECTS (*κ* ≈ 0.84) [31]. Albers et al. compared the ASPECTS and RAPID scores of four expert readers with diffusion-weighted imaging (DWI) results obtained following baseline CT; RAPID outperformed physicians in spotting early signs of cerebral ischemia identified by subsequent DWI [32].

Seker et al. investigated the consistency of the brainomix e-Aspects and the ASPECTS of two residents and two consultants. In contrast to the software, the residents displayed significant variation and lower internal concordance. However, the consultant and software scores were comparable [33].

In 214 patients undergoing EVT, Olive-Gadea et al. looked at the relationship between radiologist and e-ASPECTS scores and the infarct core CBV and infarct end-tidal volume as well as the long-term functional outcome. ASPECTS score was determined by a radiologist (Rx-ASPECTS) during acute stroke assessment and by Brainomix software (e-Aspects), while images were sent to RAPID software to quantify the ischemic core. The distribution of ASPECTS scores was similar according to their study. A mild and time-dependent correlation between ASPECTS and e-ASPECTS and CBV was found, with the best correlation occurring 180 min after the onset of symptoms. Only Rx-ASPECTS and e-ASPECTS were predictors of a good functional outcome, but CBV and e-ASPECTS predicted infarct volume after thrombectomy in a similar manner [34].

Chriashkova et al. looked at how well e-ASPECTS improved concordance with the ASPECTS reference standard and sped up the time it took to evaluate CT scans. Twenty-six clinicians with various levels of experience participated in the study. When using e-Aspects, the average time to score was reduced by 34%. All groups of clinicians who used e-ASPECTS assistance saw a twofold increase in their sensitivity to early ischemic changes, with the effects being more pronounced for less experienced clinicians [35]. The detection of LVOs has been the subject of other studies. In a cohort of 223 patients, Chung et al.’s CNN model for detecting the “hyperdense vessel sign” in the middle cerebral artery on NCCT, which is typically associated with an LVO, achieved 96% specificity [6, 36].

In a cohort of adult AIS patients with and without LVO, Barreira et al. compared the findings of a skilled neuroradiologist using CTA scans to those from Viz.ai software. The performance of Viz.ai for proximal intracranial LVOs was remarkably good [37]. For the purpose of detecting LVO in the acute setting, You et al. combined structured clinical data with unstructured CT imaging data; the evaluation system in their study contained three hierarchical models. Structured demographic and clinical data were used in the modeling’s first two levels, and a DL model’s additional CT imaging features were used in the third level. The third level of evaluation with the clinical and imaging features produced the best model performance on the test group; the accuracy, sensitivity, and area under the curve (AUC) were greater than 0.80 [38].

## Tissue outcome

To decide which patients would benefit from thrombolysis or another attempt in the event of partial recanalization, it is essential to predict final infarct volumes. To predict final infarct volume directly from native CTP images and metadata like time parameters and treatment, Robben et al. used a deep neural network. They forecasted the hypothetical final infarct volume for each test subject in the scenarios of early complete recanalization (mTICI 3 at 60 min) and in the absence of recanalization. With a mean volume error of 2.8 ml and a mean absolute volume error of 36.7 ml, the results were satisfactory [39].

By using pseudo-continuous arterial spin labeling (pCASL), Wang et al. created and assessed a DL-based algorithm to help identify AIS patients who would benefit from endovascular therapy. The outcomes showed that the algorithm had a 92% accuracy rate and a 0.94 AUC [40].

To forecast the tissue result Nielsen et al. trained a deep CNN (CNNdeep) that outperformed competing approaches and was remarkably consistent with the final result as determined by T2-FLAIR measurements. They also developed CNNdeep,rtpa, which was used to assess patients who had received intravenous rtPA. The AUC for patients who had received intravenous rtPA evaluated with CNNdeep,rtPA was 0.85 ± 0.15 [41].

Without knowing the status of reperfusion, Yu et al. used a DL model trained with acute and follow-up image collection to predict the size and location of infarct lesions at 3–to 7 days after baseline. Three to seven days after the baseline, infarct lesions could be accurately predicted by their model. Comparable performance was shown by the model in patients with and without reperfusion [42].

## Clinical outcome

Several studies looked into the use of AI to forecast stroke patients’ outcomes. Extreme gradient boosting and gradient boosting machine, which are decision tree-based algorithms, were used by Xie et al. to predict the 90 day modified Rankin scale (mRS) > 2 with AUCs greater than 0.745; performance improved when the National Institutes of health stroke scale (NIHSS) at 24 h and recanalization outcomes were taken into account [43, 44].

Bacchi et al. investigated the use of DL algorithms in predicting outcomes in patients who received r-TPA; a positive outcome was defined as the outcome of mRS 0–1 at 90 days (vs ≥ 2) (“mRS90”), or NIHSS improvement by ≥ 4 points at 24-h (NIHSS24). An AUC of 0.75 for the prediction of mRS90 and 0.70 for the prediction of NIHSS24 were obtained using CNN + ANN [45].

To identify patients who would benefit from thrombectomy and forecast both immediate and long-term clinical/functional outcomes, Tang et al. built ML logistic regression models. The mRS was used to evaluate clinical outcomes at 7 and 90 days, respectively. They combined early clinical data with the initial preprocedural diffusion and perfusion-weighted MRI datasets to create a combined model. This model was contrasted with two others that utilized clinical data alone and clinical data along with penumbra (mismatch) data. Their combined model was the most accurate at forecasting both short- and long-term clinical outcomes, with an AUC of 0.863 [6, 46].

In order to predict outcome measures (mRS ≤ 2 at 90 days) and good reperfusion (mTICI ≥ 2*b*), Hilbert et al. used DL techniques trained on CTA data. For this purpose, a number of artificial intelligence techniques were evaluated. The findings revealed that automated radiological image analysis using data-efficient DL methods outperformed the combination of multiple radiological image biomarkers for the prediction of a favorable stroke outcome. For functional outcome and reperfusion, DL models outperformed models using conventional radiological image biomarkers [47].

The accuracy of an algorithm based on functional imaging to predict deficits in various areas, including attention, visual memory, verbal memory, language, motor, and visual, was tested by Siegel et al. Lesion location performed better than functional connectivity in predicting verbal and visual memory deficits, while functional connectivity performed better than lesion location in predicting visual and motor deficits [48, 49].

A regression tree model was created by Alawieh et al. to predict mRS scores after 90 days in patients receiving ET. With a 0.952 AUC, the model successfully predicted the functional independence rates at 90 days with 89.36% sensitivity and 89.66% specificity. The outcomes far outperformed those of the NIHSS and the ASPECTS [50].

Hofmeister et al. demonstrated that a small subset of nine radiomic features was predictive of the success of first-attempt recanalization with thromboaspiration (AUC = 0.88); 4/9 radiomic features were positively associated with first-attempt recanalization after thromboaspiration (*P* < 0.05), including large area low gray level emphasis, gray level variance, large dependence emphasis, and short run emphasis; the other 5 were negatively associated (*P* < 0.05): entropy, maximum, run percentage, coarseness, and gray level nonuniformity normalized; additionally, it was demonstrated in their study that characteristics like higher HU values, texture randomness, coarseness, and clot heterogeneity were associated negatively with rapid recanalization [51]. The effectiveness of Diffusion Tensor Imaging (DTI) in predicting clinical outcomes has been examined in some studies. The functional outcome was evaluated at 3 months and was divided into two categories: good outcome (mRS ≤ 2) and poor outcome (mRS > 2). Moulton et al. retrospectively evaluated patients with AIS who received thrombolysis within 4.5 h of stroke onset and who underwent a DTI sequence at 24 h after stroke. The second and third branches of the superior longitudinal fasciculus (SLF), the corpus callosum, the corticospinal tract, the long, anterior, and posterior segments of the arcuate fasciculus (AF), and the frontal aslant tract were the regions that had the greatest influence on functional outcome [52, 53].

In other studies, the prognostic value of functional magnetic resonance imaging (fMRI) was examined to predict clinical outcomes in stroke patients at 4–6 months; 86% of patients had outcomes that their model correctly predicted [53, 54].

## Stroke complications

The ability to predict complications can have a big clinical impact. To predict cerebral edema, changes in cerebrospinal fluid (CSF) dynamics were taken advantage of. 155 stroke patients’ CSF volumes, as determined by serial CT imaging, were examined using a random forest model. They proposed that variations in CSF volume over time might serve as a quantitative indicator of edema development. In addition, a correlation between infarct volume and the decrease in CSF volume between baseline and final CT was found (*R* = 0.715) [55].

Hemorrhagic transformation should also be taken into account or predicted as a complication. Yu et al. used a variety of DL and ML models, using follow-up gradient echo MRI performed at 24 h in comparison to diffusion- and perfusion-weighted MRI as the ground truth for the hemorrhage. The most accurate (84%) model was their kernel spectral regression model [56]. With the aid of a surface neural network, Wang et al. were able to predict intracranial hemorrhage (ICH) in AIS patients receiving thrombolysis with an AUC of 0.82. Age, sex, baseline NIHSS data, admission blood pressure and glucose, prior medical history, and smoking status were all taken into account; about 50% of patients were categorized as low-risk, and none of them experienced ICH in the prospective cohort [57, 58].

## Main available software

In this Table 1, the main available software and their application were summarized.

**Table 1:** A summary of the main available AI software and their application in stroke

Software	Application	Imaging
Brainomix	*e-Aspect*: automatically assess ASPECTS score *e-CTA*: assessment of collaterals and LVOs *e-CTP*: automatically estimates core and penumbra volumes, in addition to mismatch ratio and HIR	NCCT, CTA, CTP
Rapid	*Rapid ICH*: identifies and classifies ICH *Rapid ASPECTS*: automatically assess ASPECTS *Rapid CTA*: assessment of blood density asymmetry *Rapid CTP*: automatically delivers quantified and color-coded CT perfusion maps *Rapid MRI*: automatically delivers advanced MR diffusion and perfusion image analysis	NCCT, CTA, CTP, MRI
Viz.ai	*Viz LVO*: automatically detect and alert emergency stroke team *Viz CTP*: automatically analyze CTP images of brain, calculating CT perfusion parameters *Viz ICH*: detection of ICH	NCCT, CTA, CTP

### Comparison between software

**Fig. 1:**
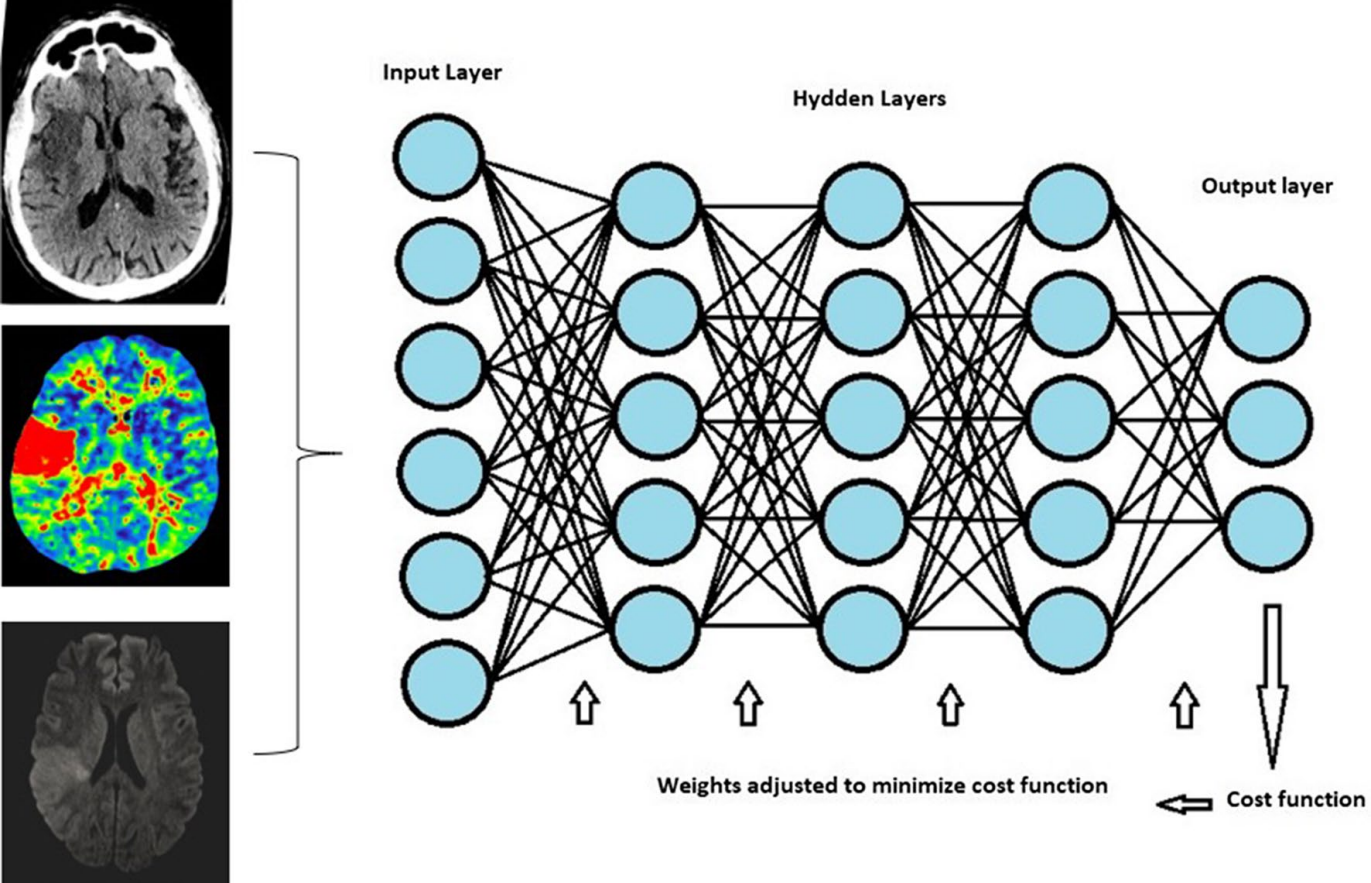
An illustration of artificial neural networks, a branch of ML. The input layer is the first layer, followed by a configurable number of hidden layers, and an output layer. Each layer is made up of nodes or neurons

**Fig. 2:**
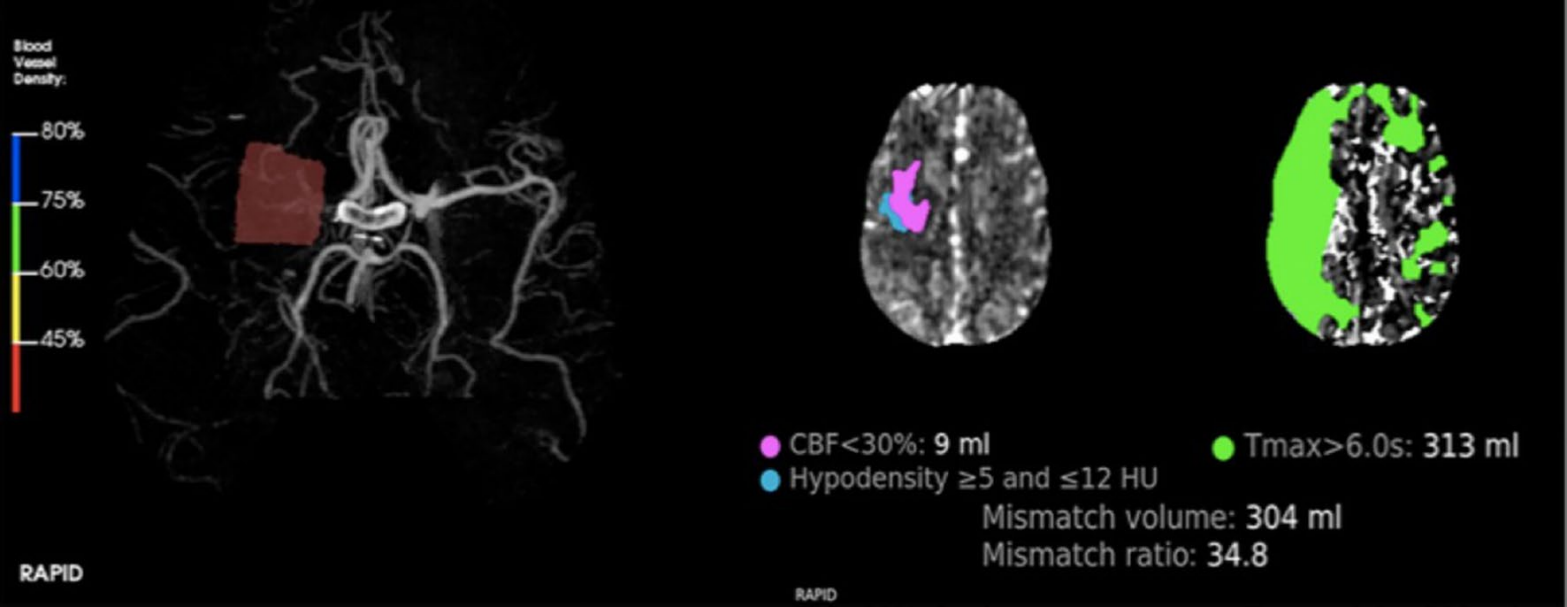
Processing performed by RAPID—LVO software showing flow reduction (vessel density < 45% in right M1); RAPID software also calculates CBF and T max for volume and mismatch ratio, crucial for endovascular treatment planning

It is demonstrated that the performance of Brainomix’s e-ASPECTS is superior to or on par with that of humans (Fig. 1). Rapid similarly scores the ASPECTS, identifies LVOs, and assesses collaterals by examining the asymmetry of the contrasted vessels. e-CTA software provides a quick assessment of LVOs and performs a collaterals estimation. Additionally, RAPID automates the assessment of stroke patients’ suitability for thrombectomy (Fig. 2). Each program has the capacity to produce data, including CT and MR perfusion maps [59].

**Fig. 3:**
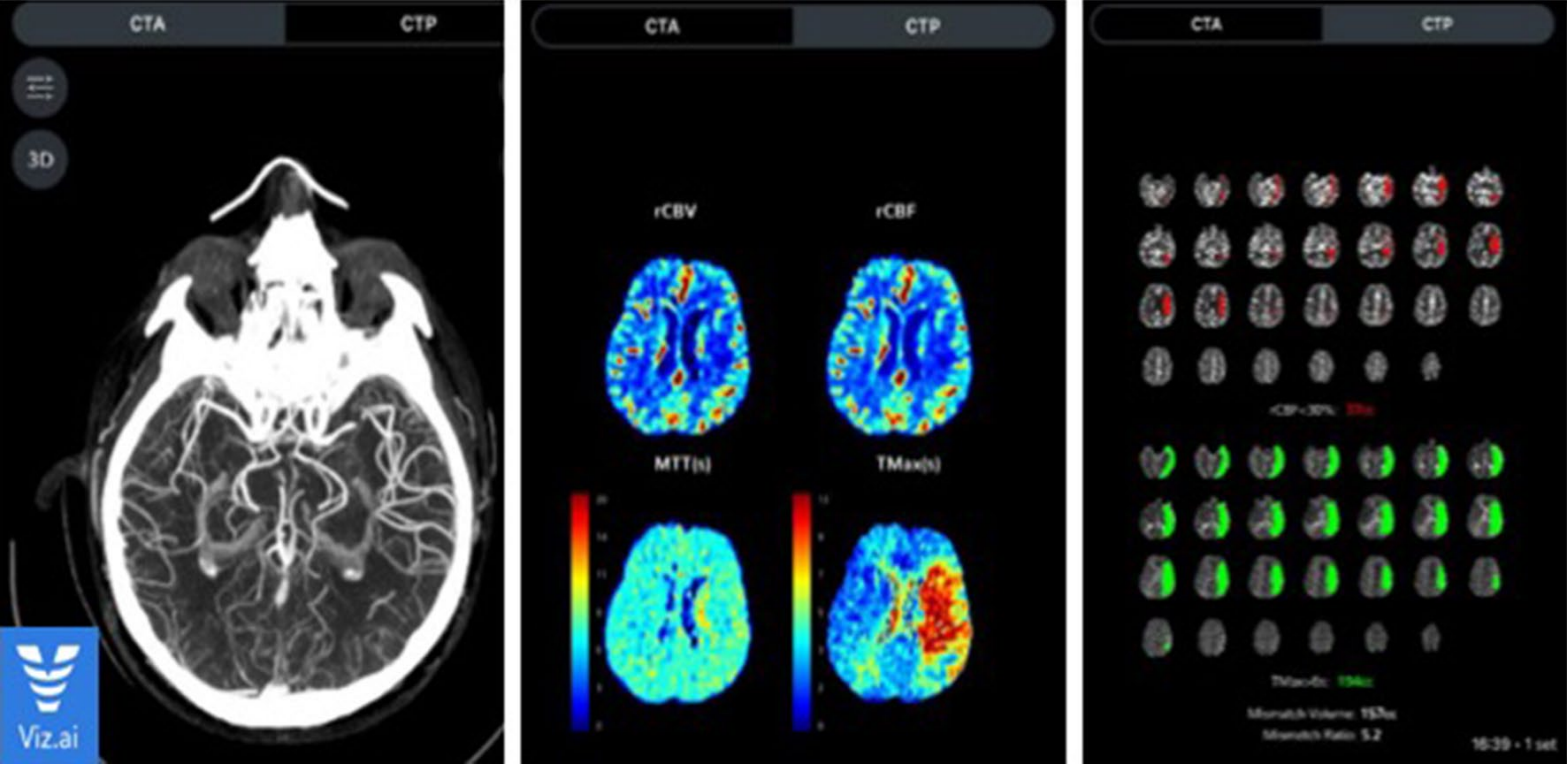
An example of Viz.ai data processing; in this case LVO is detected and software automatically alerts the stroke team

Hoelter et al. found high correlation between Brainomix and RAPID median ASPECTS: *r* = 0.835 (0.512, 0.923), *P* < 0.001. While there are some differences, such as automatic motion correction capabilities and the automatic activation of the stroke team, Viz.ai shares some characteristics with the software mentioned above (Fig. 3) [59, 60].

## Potential and limits

AI has demonstrated a positive effect on stroke by making components like ASPECTS immediate and immune to inter-individual variation. An intriguing aspect of clinical practice is that helping neuroradiologists by performing a preliminary image analysis has the same overall impact as what the residents do for the signatory physician’s medical doctor—a reduction in the time between diagnosis and treatment. Limitations like false positives demonstrate the importance of the neuroradiologist’s assessment for accurate diagnosis. Other drawbacks might include the fact that the ML algorithm does not perform as well in patients with strokes who have abnormal brain features, as demonstrated by Gueberina et al. [61, 62].

The development of software that can automatically detect and/or segment the thrombus after it has been highlighted must take into account the possibility that in clinical practice the cohort of patients to whom this program would then be applied may present some discrepant characteristics with respect to the study group. For example, the developers must take into account the presence of calcifications, moveable thrombi, and other factors before considering using the programs in a commercial setting [63].

## Ethical and legal issues

There are some medico-legal issues that cannot be disregarded when it comes to the use of AI in the medical and, in this case, radiological fields. There are numerous issues with deciding which AI algorithms or medical devices to approve for use in the U.S. and Europe, particularly for those that might not require human intervention. The majority of advancements in this area focus on developing tools that can support physicians rather than replace them [64]. Liability is still up for debate in the event that AI software causes a medical error. Currently, it is the physician’s responsibility to ensure that the algorithm’s output complies with diagnostic standards [53].

## Economic impact

Due to the severe impairment it causes, stroke has a significant financial impact on the healthcare systems. Each minute of thrombectomy delay results in roughly a 4-day loss of disability-free life, according to the study Highly Effective Reperfusion Evaluated in Multiple Endovascular Stroke Trials (HERMES) [65].

The average cost of thrombectomy delay is roughly $1059 per minute; if 10 min could be avoided on average in the USA, this would result in an annual savings of $249 million [66]. The great benefit of available software is that it ensures the least amount of time is spent on patient management. Hassan et al. compared transfer times before and after the implementation of the Viz.ai system for all LVO patients who were transferred from the spoke (PSC) to their hub center (HC). The median transfer times from the PSC to the HC were cut in half by 22.5 min, and from the CTA to the PSC to the puncture to the HC were cut in half by 89 min [67]. Reducing time from diagnosis to treatment has a profound effect on the amount of money saved each year.

## Current state of AI software in AIS and future directions

As was already mentioned, current commercial software focuses on the detection of AIS and estimation of the key characteristics that are essential to understand during the emergency phase, such as collaterals, ASPECT, and perfusion parameters [30–35].

Future scientific research, however, is moving in a direction that includes the study of additional elements that could soon be used in clinical practice, such as, for example, the use of radiomics and advanced imaging parameters for the correlation with the patient’s long-term deficit; this scenario opens up new possibilities for the application of therapeutic pathways in the context of a personalized medicine [51].

## Conclusion

As was already mentioned, current commercial software focuses on the detection of AIS and estimation of the key characteristics that are essential to understand during the emergency phase, such as collaterals, ASPECT, and perfusion parameters. Future scientific research, however, is moving in a direction that includes the study of additional elements that could soon be used in clinical practice, such as, for example, the use of radiomics and advanced imaging parameters for the correlation with the patient’s long-term deficit; this scenario opens up new possibilities for the application of therapeutic pathways in the context of a personalized medicine. AI will undoubtedly take up more and more space in both research and hospitals.
